# Comparison of miRNA quantitation by Nanostring in serum and plasma samples

**DOI:** 10.1371/journal.pone.0189165

**Published:** 2017-12-06

**Authors:** Catherine Foye, Irene K. Yan, Waseem David, Neha Shukla, Yacob Habboush, Lori Chase, Kristen Ryland, Vivek Kesari, Tushar Patel

**Affiliations:** Department of Transplantation, Mayo Clinic, Jacksonville, Florida, United States of America; Saint Louis University, UNITED STATES

## Abstract

Circulating microRNAs that are associated with specific diseases have garnered much attention for use in diagnostic assays. However, detection of disease-associated miRNA can be affected by several factors such as release of contaminating cellular miRNA during sample collection, variations due to amplification of transcript for detection, or controls used for normalization for accurate quantitation. We analyzed circulating miRNA in serum and plasma samples obtained concurrently from 28 patients, using a Nanostring quantitative assay platform. Total RNA concentration ranged from 32–125 μg/ml from serum and 30–220 μg/ml from plasma. Of 798 miRNAs, 371 miRNAs were not detected in either serum or plasma samples. 427 were detected in either serum or plasma but not both, whereas 151 miRNA were detected in both serum and plasma samples. The diversity of miRNA detected was greater in plasma than in serum samples. In serum samples, the number of detected miRNA ranged from 3 to 82 with a median of 17, whereas in plasma samples, the number of miRNA detected ranged from 25 to 221 with a median of 91. Several miRNA such as miR451a, miR 16-5p, miR-223-3p, and mir25-3p were highly abundant and differentially expressed between serum and plasma. The detection of endogenous and exogenous control miRNAs varied in serum and plasma, with higher levels observed in plasma. Gene expression stability identified candidate invariant microRNA that were highly stable across all samples, and could be used for normalization. In conclusion, there are significant differences in both the number of miRNA detected and the amount of miRNA detected between serum and plasma. Normalization using miRNA with constant expression is essential to minimize the impact of technical variations. Given the challenges involved, ideal candidates for blood based biomarkers would be those that are indifferent to type of body fluid, are detectable and can be reliably quantitated.

## Introduction

The potential for circulating miRNA to serve as biomarkers has stimulated a wide range of investigation regarding the disease-specific expression and stability of expression [1]. Detection of circulating miRNA has been performed using PCR based approaches. Recently the Nanostring nCounter platform has been introduced in clinical laboratory settings for detection, quantitation and assessment of gene expression. It offers the advantage of direct quantitation in blood samples, thereby avoiding potential biases towards abundant miRNA that may arise from RNA amplification [2, 3]. This platform is FDA approved and currently in use for testing gene signatures such as the PAM50 gene signature to assess the risk of recurrence and patient stratification in breast cancer in postmenopausal women [4]. Thus, diagnostic assays using this platform could be translated to a clinical setting.

In order to develop meaningful diagnostic assays based on circulating miRNA, detection and quantitation has to be standardized, consistent and reproducible, and the assays should have sufficient sensitivity and precision. The development of any test is subject to pre-analytical factors such as specimen collection and processing that can impact on test performance [5]. Detection of miRNA can be influenced by the type of blood sample, collection protocol, extraction and detection methods [6–9]. In this study, we performed expression profiling of 798 miRNA in blood samples using the Nanostring platform to examine differences in detection and quantitation of miRNA between paired concurrently obtained serum and plasma samples, and evaluated appropriate normalization techniques.

## Materials and methods

### Blood sample collection and processing

The samples used for Nanostring analysis included matched serum and plasma samples from 28 patients. Written informed consent was obtained from participants under an IRB approved protocol (#14–4570). The blood samples were prospectively collected at the same time from each patient and following a defined standard operating procedure for collection and processing. Samples were collected between August 1, 2015 and June 30, 2016. Blood was collected from a peripheral vein using a 21 gauge needle. Samples were transferred or collected in BD vacutainer serum tubes or plasma tubes. The Mayo Clinic-Florida Biospecimens Accessioning and Processing lab processed all samples within 2 hours of collection. Samples were spun at 2900*g* for 10 minutes at 4 degrees Celsius. Samples were then aliquoted into micro centrifuge tubes and stored at -80 degrees Celsius. Plasma was withdrawn without disturbing the buffy coat and immediately stored at -80C in 500μl aliquots. For serum collections, blood was allowed to clot for 30 minutes at 4°C and centrifuged at 3000 rpm for 10 minutes at 4°C. Prior to analysis, samples were thawed on ice.

### RNA isolation and purification

Blood samples were thawed on ice. 500μl was transferred into a new tube and spun at 3,000 x *g* for 5 minutes to pellet any cells. Serum was transferred to a new tube and RNA was isolated using miRCURY RNA isolation kit (Exiqon, Denmark) following manufacturer’s instructions including the on-column DNase treatment. Next, the sample was concentrated using the RNA Clean and Concentrator-5 kit (Zymo Research Corp., Irvine, CA) and eluted in 15μl of water. RNA was analyzed using NanoDrop (Thermo Scientific, Waltham, MA).

### RNA expression analysis

Analysis was performed on all samples using the nCounter Analysis System (NanoString Technologies) and the nCounter Human v2 miRNA Panel which contains 798 unique miRNA barcodes. Probes for several Housekeeping genes such as Ribosomal protein L10 (RPL10), beta-actin (ACTB), beta-2-microglobulin (B2M), glyceraldehyde 3-phosphate dehydrogenase (GAPDH), and ribosomal protein L19 (RPL19), as well as for endogenous miRNAs for Arabidopsis thaliana miR159a (ath-miR159a), Caenorhabditis elegans (cel)-miR-248 and miR254, and Oryza sativa (osa)-miR 414 and 442 are incorporated in the Nanostring codesets and were used for analysis along with positive and negative controls. RNA was prepared and run according to the manufacturer’s protocol in a total of 6 runs over 8 days. To test for RNA degradation, several sentinel samples were examined by Bioanalyzer 2100. RNA was loaded at 100 ng per sample, and no low-count quality control flags were observed for any of the samples. All hybridizations were 18 hours long, and all counts were gathered by scanning on HIGH mode for 280 fields of view per sample.

### nCounter data analysis

Analysis of raw miRNA data was performed using the NanoString technologies nSolver analysis software version 3.0. Background correction was performed by subtracting the mean + 2 standard deviations of negative control as a cut-off. Analyses of stability of expressed miRNA was performed for miRNA with average counts above background using geNorm [10]. A count of 5 or more after background correction was used to define the presence of a given miRNA. For technical variations, code-set content normalization was performed with the geometric median for all genes.

## Results

### Samples and RNA yield

Serum and plasma samples were from 28 patients, 12 male and 16 female (Table 1). The age range was from 40–80 years, with a median age-range of 64 years. 3 patients had benign disease (Non-alcoholic steatohepatitis or primary sclerosing cholangitis), whereas 25 patients had primary liver cancer. Of these, 16 patients had cholangiocarcinoma whereas 9 had hepatocellular cancer. Both serum and plasma samples were collected at the same time from a single venipuncture and processed using a standard protocol. RNA was isolated from aliquots of serum and plasma, and profiling for 798 miRNA was performed using Nanostring. The RNA concentration obtained ranged from 32–125 μg/ml from serum and 30–220 μg/ml from plasma samples.

**Table 1:** Demographics of the study population.

		Total (n = 28)	Male (n = 12)	Female (n = 16)
Age	Median (years)	64	61	66
	Range (years)	40–79	58–76	40–79
Cancer	Cholangiocarcinoma	16	6	10
	Hepatocellular cancer	9	5	4
Etiology	Alcohol	6	3	3
	HCV	2	1	1
	NASH	3	2	1
	PSC	2	0	2

Demographics of study population. HCV: Hepatitis C; NASH: Non-alcoholic steatohepatitis; PSC: Primary sclerosing cholangitis

### Comparison of miRNA detection in serum or plasma

**Fig 1:**
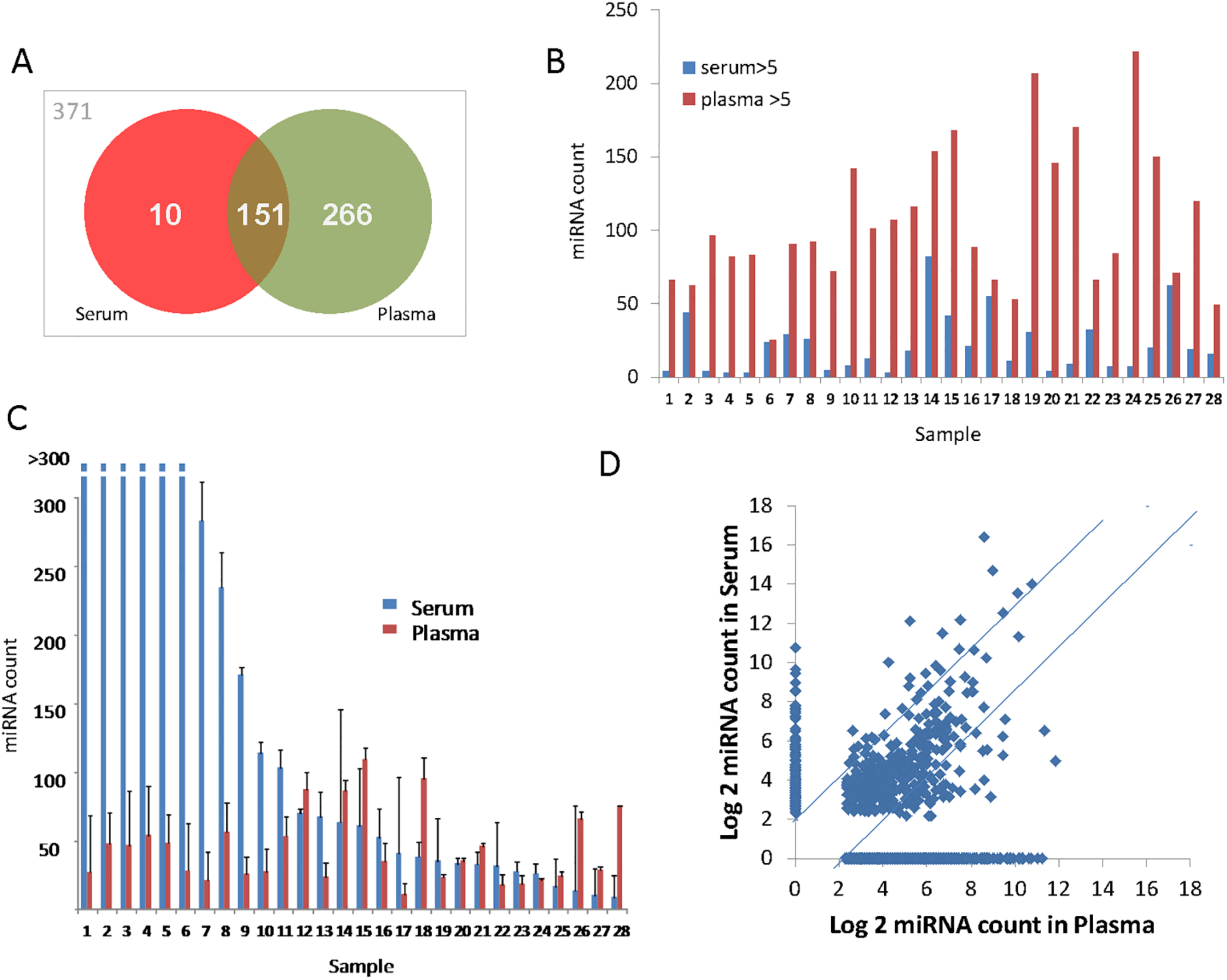
miRNA counts differ in serum and plasma. A. Presence of circulating miRNA detected by Nanostring in serum and plasma. Of the 798 miRNA examined, 371 were not detected at levels above background, whereas 427 were detected in serum, plasma, or in both serum and plasma. B. The number of individual miRNA detected with counts >5 after background correction are shown for serum or plasma from each subject. C. Quantitative expression of miRNA in plasma and serum. Data represents average and standard deviation of all detected miRNA, with counts greater than 5 after background correction. D. Correlation of all individual miRNA counts in plasma and serum.

The serum and plasma miRNA spectrum in serum and plasma was profiled using Nanostring assays, and quantitation performed after background correction and median normalization. Individual miRNA counts differed considerably in plasma and serum. The results are depicted as scatter plots (Fig 1). There were differences noted between the specific miRNA detected (counts greater than 5) as well as the counts of each miRNA between serum and plasma. A larger number of individual miRNA were detected in plasma than in serum. However, miRNA counts were greater in serum than in plasma for most of the samples. In serum samples, the number of detected miRNA ranged from 3 to 82 with a median of 17, whereas in plasma samples, the number of miRNA detected ranged from 25 to 221 with a median of 91. After background correction, the average count in serum was 19.44 whereas in plasma was 19.14. Of note, there were 10 miRNA that were detected in serum but not plasma samples, and 266 miRNA that were detected in plasma but not in serum samples. Of the 798 miRNA, 371 miRNA were not detected in any of the samples tested, 427 detected in either serum or plasma, whereas 151 miRNA were detected in all of the samples tested (Fig 1). In contrast to the marked differences in miRNA detection between serum and plasma, we did not observe any significant correlation between the number of detectable miRNA species and the gender of the donor, the type of sample used (serum or plasma), or the RNA concentration.

### Utility of endogenous and exogenous controls

**Fig 2:**
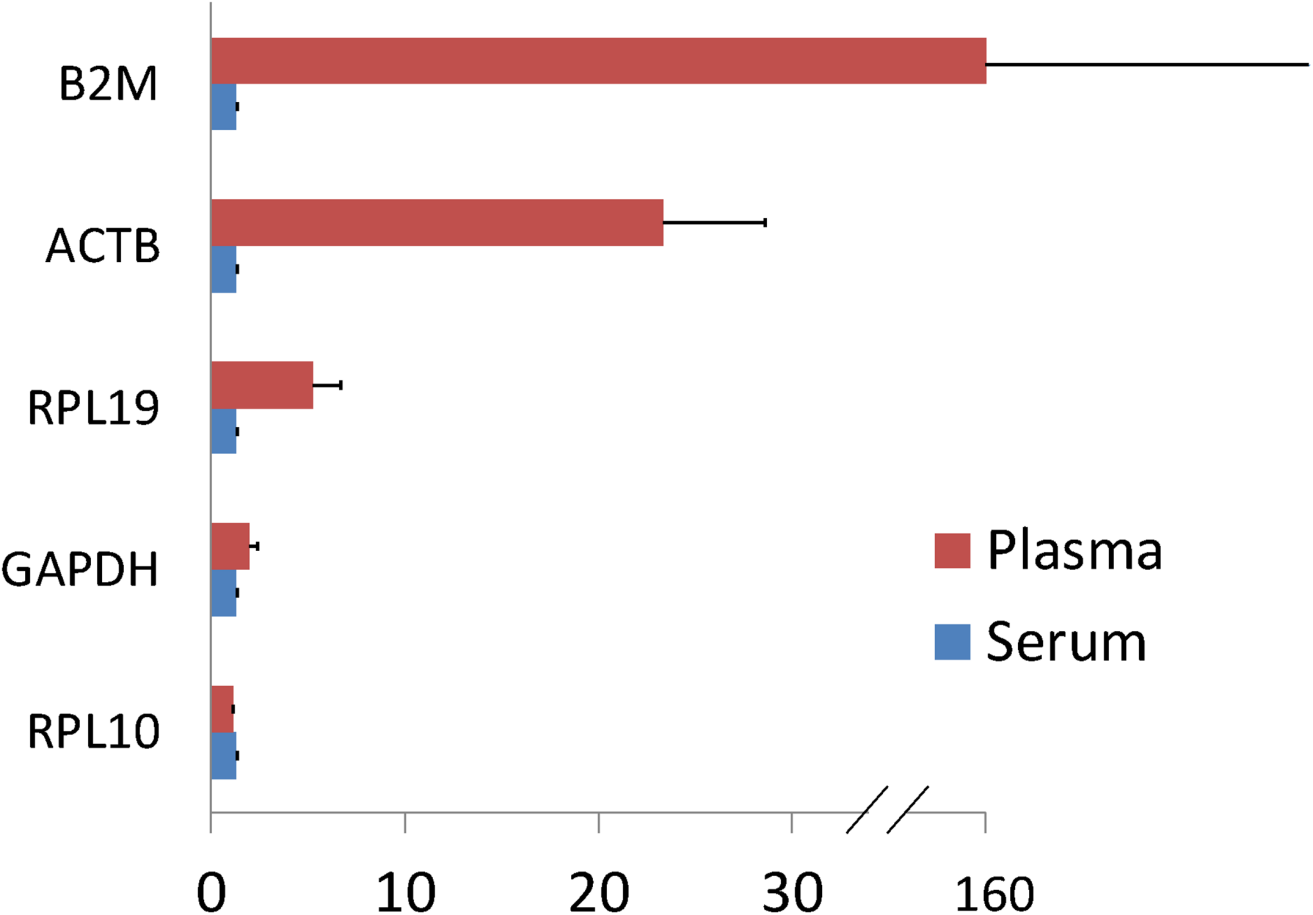
Expression of putative endogenous controls. Expression in serum and plasma of endogenous mRNA used as normalizers. Average count and SD from 28 individual samples for each group are shown. ACTB, beta-actin; B2M, beta-2-microglobulin; GAPDH, glyceraldehyde 3-phosphate dehydrogenase; RPL10, Ribosomal protein L10; RPL19, ribosomal protein L19.

We next analyzed the quantitation of a series of putative endogenous controls: ACTB, B2M, GAPDH, RPL19, and RPL10. Measurements in serum revealed low expression of each one of these (Fig 2A). However, there was considerable variation noted in their quantitation from plasma samples. While RPL10 and GAPDH were low abundance and below background levels, their expression counts were consistent with samples. In contrast, B2M, ACTB and RPL19 had counts>5 after background correction in serum and/or plasma, and also varied between serum and plasma. A comparison of average counts for each of these is shown in Fig 2. The greatest variation was noted for B2M, a putative tumor marker that is also increased in liver disease. An average B2M count of 208 was noted in cancer plasma samples compared with an average count of 12 in non-cancer plasma samples. These results indicate the need for caution when one or more of these are used to normalize expression levels of miRNA across different samples, or across laboratories.

**Fig 3:**
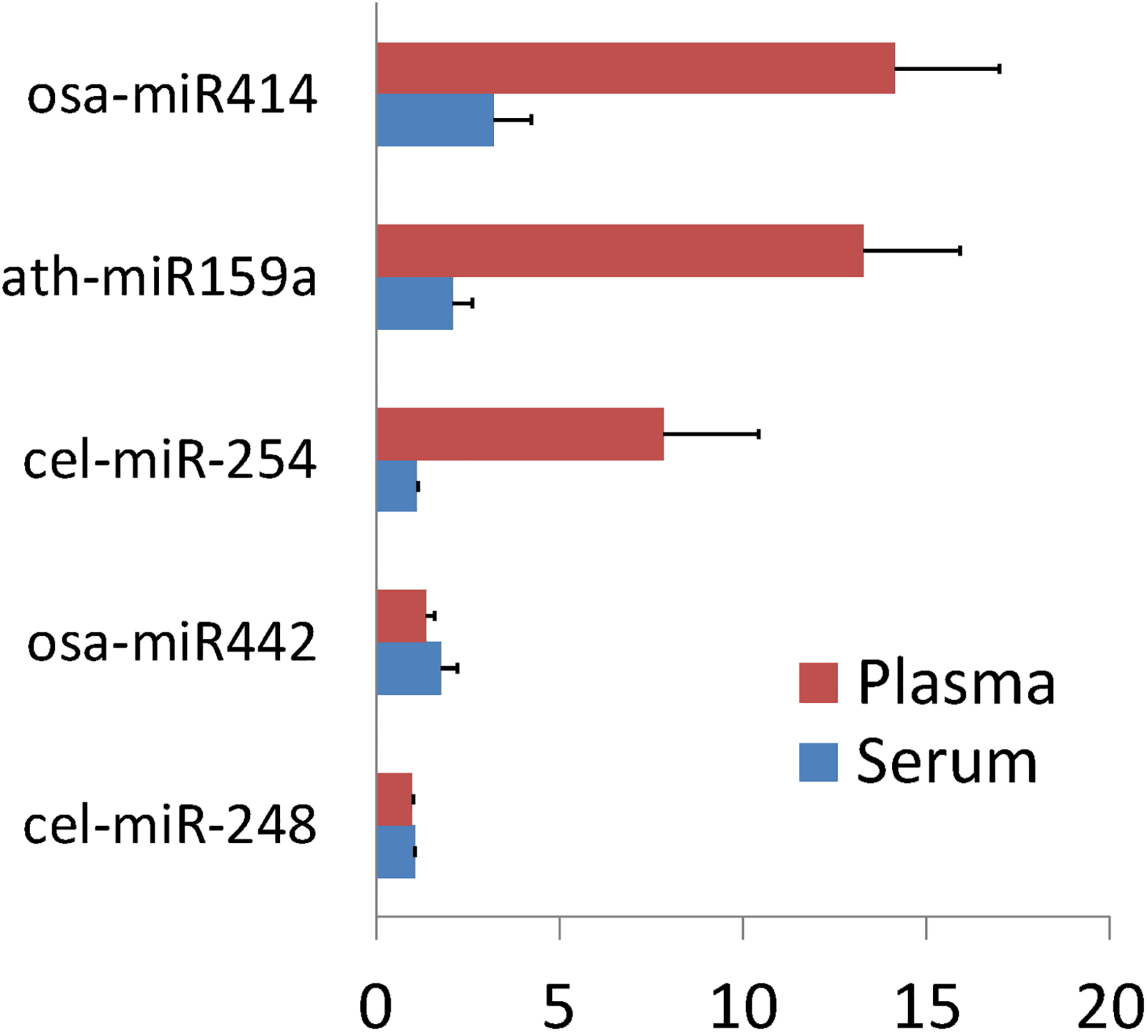
Basal expression of putative exogenous controls. Serum and plasma expression of exogenous small non-coding RNA from Arabidopsis thaliana, Caenorhabditis elegans and Oryza sativa, in the absence of input RNA. Average count and SD from 28 individual samples are shown. ath, Arabidopsis thaliana miR; cel, Caenorhabditis elegans; osa, Oryza sativa.

We next examined the detection of expression of non-endogenous miRNA, ath-miR159a, cel-miR-248 and miR254, and osa-miR 414 and 442. The average count of all negative controls was 13.8. Similar to observations made with endogenous controls, levels in plasma samples were greater that those from serum samples (Fig 3). Under the presumption that these exogenous miRNA should not be present in human circulation, these results may result from a false positivity, or reduced specificity for detection of exogenous small non-coding RNA using Nanostring in plasma samples.

### Most stably expressed miRNA in both serum and plasma

We next sought to identify miRNA that were most stably expressed across samples, as candidates for internal normalization. Gene expression stability was performed using geNorm to derive geometric means using raw miRNA counts. Out of all miRNAs, the top ten ones with the least variation using geNorm and raw counts are shown in Table 2.

**Table 2:** Gene expression stability.

miRNA	MC < 1.5	Serum		Plasma	
		Average count	SD	Average count	SD
hsa-miR-95-3p	0.731	1.3	0.052	3.221	0.857
hsa-miR-92b-3p	0.736	1.286	0.052	1.321	0.195
hsa-miR-576-5p	0.737	1.286	0.052	2.041	0.503
hsa-miR-511-5p	0.74	1.286	0.052	1.406	0.154
hsa-miR-608	0.744	1.286	0.052	1.225	0.09
hsa-miR-876-3p	0.75	1.286	0.052	2.002	0.452
hsa-miR-579-5p	0.75	1.286	0.052	1.132	0.027
hsa-miR-337-3p	0.754	2.925	0.758	17.445	2.625
hsa-miR-363-5p	0.755	1.286	0.052	1.196	0.047
hsa-miR-378f	0.755	1.653	0.192	13.554	2.866

Gene expression stability was performed using geNorm to derive geometric means using raw miRNA counts from all serum and plasma samples. The gene-stability measure *M* is the average pairwise variation between this miRNA gene and all other genes. An increasing ratio variation corresponds to decreasing expression stability of the tested gene. The top ten most highly stable miRNA with least variation on expression in all samples are shown, along with corresponding average counts and SD in serum and plasma.

Gene expression stability could be skewed by data from miRNA that are low abundance and beyond the sensitivity of detection, or are not present in the sample. We therefore examined expression stability testing on miRNA with counts greater than 5 following background correction in either serum or in plasma samples. Although the top most stable miRNA differed between serum and plasma, this analysis identified miR-30e-5p as being the least variable in either serum or plasma (Table 3).

**Table 3:** Expression stability of all detectable miRNA.

miRNA	Serum			miRNA	Plasma		
	M	Average Count	SD		M	Average Count	SD
hsa-miR-30e-5p	0.878	30	14	hsa-miR-30e-5p	0.927	39	28
hsa-miR-548y	0.922	23	9	hsa-miR-1255a	0.936	23	21
hsa-miR-584-3p	0.925	23	8	hsa-miR-597-5p	0.942	28	24
hsa-miR-378h	0.926	23	10	hsa-miR-337-3p	0.947	41	33
hsa-miR-891a-5p	0.927	24	8	hsa-miR-497-5p	0.961	36	31
hsa-miR-1255a	0.928	22	8	hsa-miR-455-5p	0.969	34	33
hsa-miR-376a-3p	0.930	30	11	hsa-miR-626	0.969	33	33
hsa-miR-888-5p	0.933	23	9	hsa-miR-514b-5p	0.974	32	27
hsa-miR-497-5p	0.936	23	9	hsa-miR-378f	0.975	36	34
hsa-miR-548g-3p	0.951	32	13	hsa-miR-185-5p	0.976	27	22

Gene expression stability was performed using geNorm to derive geometric means using background corrected miRNA counts from serum or plasma samples. The gene-stability measure *M* is the average pairwise variation between this miRNA gene and all other genes. An increasing ratio variation corresponds to decreasing expression stability of the tested gene. The average counts and SD of the top ten most highly stable miRNA with least variation on expression in either serum or in plasma are shown.

**Fig 4:**
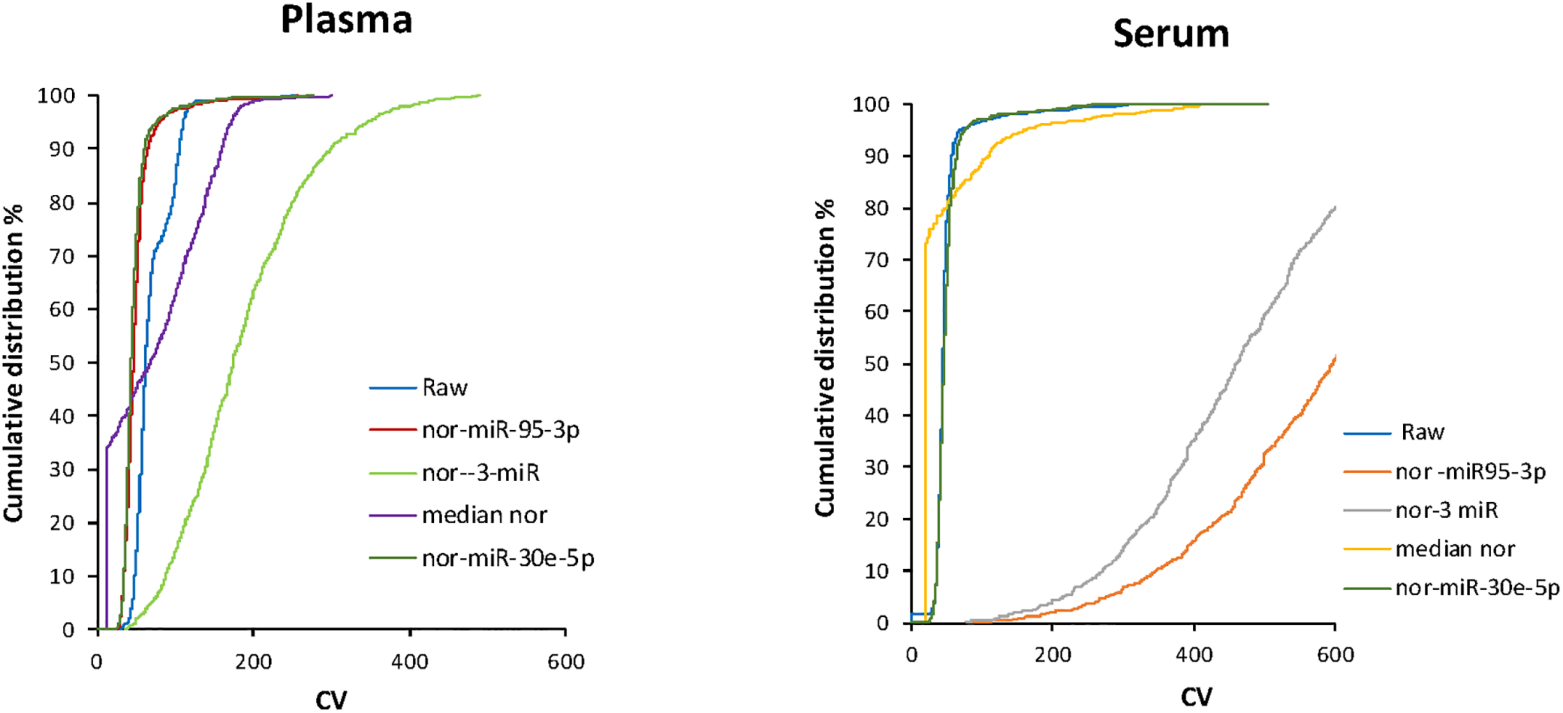
Cumulative distribution of miRNA coefficient of variation (CV) values. The cumulative distribution of the individual CV values for all expressed miRNA in the plasma and serum sets was plotted for raw (not normalized) data, and for normalized (nor) data using the most stable small RNA (miR-95-3p), mean values of the three most stable expressed miRNA (-3-miR), miR-30e-5p, or median normalization.

These miRNA could be useful as endogenous controls, either singly or in combination. We further examined the impact of using these miRNA for normalization. The cumulative distribution of the individual CV values was plotted for both raw (not normalized) and normalized data using the most stable miRNA from raw data (miR 95-3p), most stable expressed miRNA (miR-30e-5p), the three most stable expressed miRNA in either serum or in plasma, or using median normalization. Fig 4 shows the cumulative distribution of individual CV values with each of these normalization strategies compared with raw data. Median normalization provided a lower CV for serum samples, whereas for plasma samples, normalization to miR-95-3p, or to three most stable miRNA was superior to median normalization.

### Most consistent miRNA in both serum and plasma

The 10 most highly expressed miRNA with similar expression in both serum and plasma are listed in Table 4. The mean counts of these miRNA in serum ranged from 6.15 to 50.27, whereas in plasma ranged from 6.35 to 50.16. We speculate that these miRNA are least likely to be influenced by cell contamination, during clot formation or separation of plasma. Analysis of their expression in published reports from RBC, WBC or platelets revealed that they variably expressed. An appropriate strategy to identify novel biomarker candidates in discovery studies could be to limit analyses to such miRNA that are invariant and consistent in both serum and plasma. Conversely, large differences were noted between serum and plasma for several miRNA (Table 5), with the maximal difference noted for miR-451.

**Table 4:** Most highly expressed miRNA, with counts >5, that are similar in serum and plasma.

miRNA	Serum Count	Plasma Count	Serum/plasma ratio
hsa-miR-548g-3p	9.8	10.2	0.97
hsa-miR-302a-5p	6.2	6.4	0.97
hsa-miR-543	9.6	9.8	0.98
hsa-miR-128-1-5p	13.8	14.0	0.99
hsa-miR-19b-3p	40.0	40.0	1.00
hsa-miR-302a-5p	36.9	36.9	1.00
hsa-miR-585-3p	43.6	43.5	1.00
hsa-miR-544	50.3	50.2	1.00
hsa-miR-26a-5p	12.5	12.1	1.03
hsa-miR-6721-5p	24.6	23.7	1.04

The top ten miRNA that are most similar in expression between serum and plasma. The counts in each body fluid and ratio of expression are shown.

**Table 5:** miRNA with the greatest difference in counts noted in serum and plasma.

	Difference between serum and plasma	log2 difference
hsa-miR-451a	85452.18	16.4
hsa-miR-16-5p	2766.07	11.4
hsa-miR-223-3p	1016.81	10.0
hsa-miR-25-3p	1016.81	10.0
hsa-miR-4454+hsa-miR-7975	621.18	9.3
hsa-let-7b-5p	556.32	9.1
hsa-miR-191-5p	463.18	8.9
hsa-miR-126-3p	366.92	8.5
hsa-miR-873-3p	172.85	7.4
hsa-miR-144-3p	160.52	7.3

The top ten miRNA that are most dis-similar in expression between serum and plasma. The difference in counts and log 2 difference are shown.

### Confounding by blood cell miRNA release

**Fig 5:**
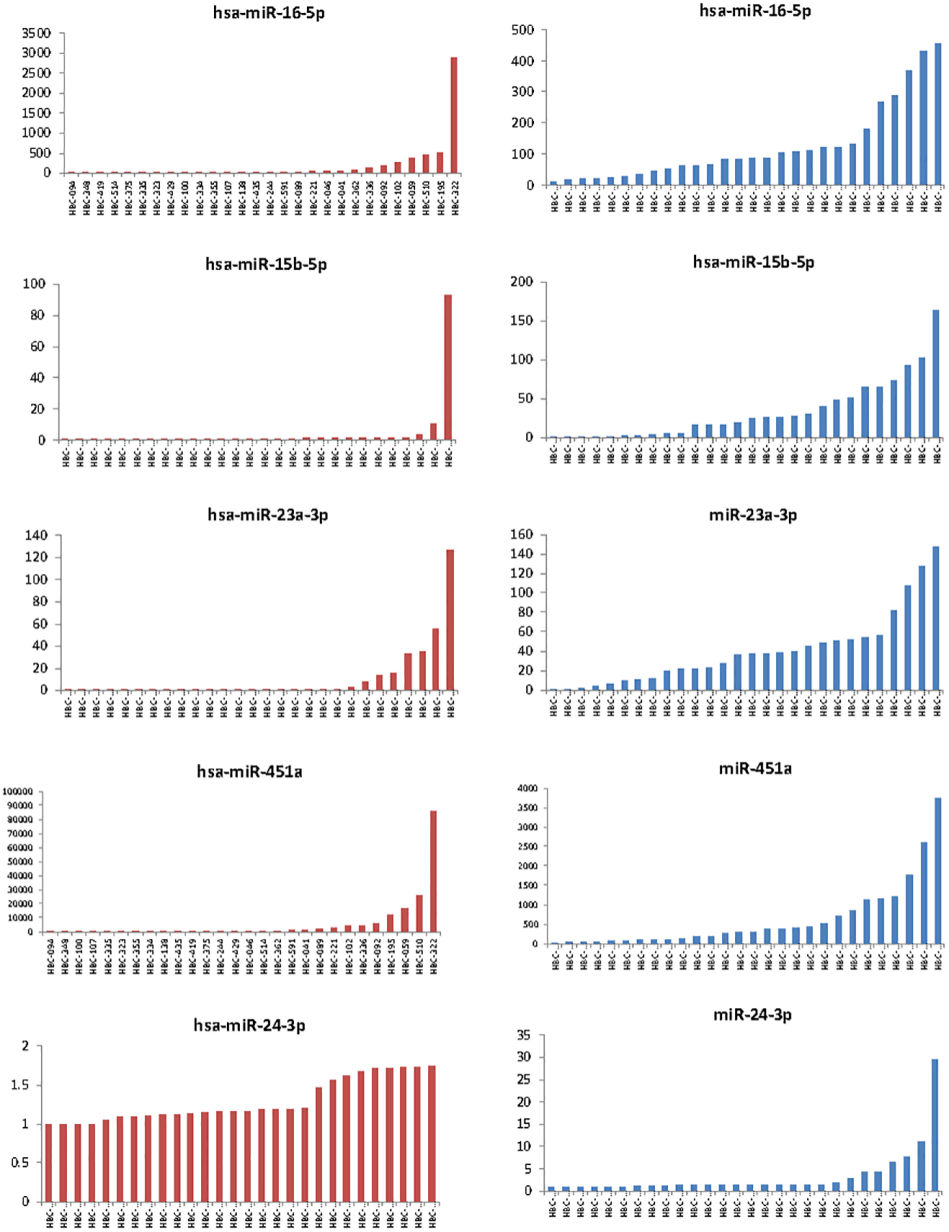
Quantitation of putative miRNA markers of contamination from blood cells. Counts of selected miRNA by Nanostring analysis from serum (left panels) and plasma (right panels).

We examined several miRNA that are expressed in blood cell components. Wang *et al*. noted that 80 of 742 miRNA could be detected in serum and plasma as well as WBC, RBC and platelets [11]. The distribution of selected miRNA (mir24-5p, miR 16-5p, mir15b-3p, and 23a-3p, and miR451a) in serum and plasma is shown in Fig 5. miR-24-5p has been shown to be unique to WBC. The average levels of miR-24-5p were higher in plasma compared with serum. Plasma miR-24-5p levels were elevated greater than 2-times the median value in 7 of 28 samples but serum levels in these were increased only in 2 of these. We postulate that these reflected release from WBC contamination in plasma. Hemolysis can result in cellular miRNA release following rupture of red blood cells. Hemolysis was not noted on direct visualization of any sample. RBC-derived miR-16-5p and miR-15b-3p have been used as surrogates for high and low abundance microRNAs, whereas miR-23a-3p has been used as a hemolysis-insensitive miRNA. The ratio of miR451a to 23a-3p evaluated by PCR has been postulated to reflect hemolysis with a greater sensitivity than colorimetric visualization and proposed as a quality control step [12]. This is based on low and constant levels of mIR23a-3p. However, using the Nanostring platform, the CV for miR-23a-3p was 64.8, with intermediate stability of expression with M = 1.26 by geNorm analysis. Further studies will need to be designed to evaluate the use of more consistently detected miRNA as denominators for the evaluation of hemolysis in samples analyzed using this platform.

## Discussion

Selection of the most appropriate type of blood sample for microRNA expression profiling is crucial because processing has the potential to influence the presence of candidate biomarkers. Plasma is obtained after centrifugation of blood that is collected in tubes containing anticoagulant, whereas serum is obtained after centrifugation of blood that has been collected and allowed to spontaneously clot in tubes without anticoagulant. During clot formation, there is release of several constituents from blood cells [13]. Blood cells can contain microRNAs that may be candidates for biomarkers. These could potentially mask detection or lead to erroneous results when they overlap with bona-fide disease markers.

In this comprehensive analysis of miRNA expression in serum and plasma using Nanostring, we identified large differences in both the miRNA detected as well as the amount of miRNA quantitated. Variations in miRNA present in serum and plasma can arise from contamination occurring due to the release of miRNA from cells. Moreover, the presence of hemolysis can alter the detectable microRNA. In the case of serum samples, clot formation can result in cell lysis and the release of miRNA enriched within blood cells and platelets. In the case of plasma samples, cells can be inadvertently aspirated during collection. The large differences observed using Nanostring are distinct from those reported using RT-PCR for detection of miRNA in a limited sample set [14]. A potential explanation for this could be that differences in the amount of very low abundant transcripts may not have been adequately detected by PCR, but were identified using Nanostring which does not rely on amplification of circulating transcripts. The important of our observations is highlighted by the findings that most biomarkers will represent low abundance transcripts in circulation.

The expression levels of miRNA can be influenced by hemolysis. The ratio of miR-451 to miR-23 assessed by PCR analysis has been postulated as a quality measure and marker of hemolysis [15]. While a ratio of >7 has been used as a marker of hemolysis, this metric cannot be directly applied for use in Nanostring analyses because expression of miRNA by PCR depends on amplification steps, and the non-linear relationship between Ct value and transcript number, Although miR-23 expression is considered insensitive to hemolysis, we observed a correlation between miR-23 and miR451 levels in our samples. While miR-23 expression was relatively stable, high amounts were observed in some cases, and associated with very high levels of miR-451. It is likely that these high values of miR-23 are not related to hemolysis in these samples.

Normalization is essential in order to minimize the impact of technical variations. This can be done using small RNA controls, or expression of selected miRNA. The use of synthetic RNA molecules as spike-in controls can correct for extraction efficiency (when added to cells prior to RNA isolation) or for reverse transcription efficiency, when added to RNA. In studies of microRNA expression based on RT-qPCR based assays, the use of mean miRNA expression values out-performed the use of endogenous small RNA controls for data normalization. Our results show that the mean value of all expressed miRNAs by Nanostring analysis is characterized by high expression stability. Thus, this approach is suitable for use in normalization and can adequately remove sources of technical variation across different code sets. While mean normalization results in reduction of noise over all expressed miRNA, stable small RNA control normalization only achieves this for the 50% least variable miRNAs. This suggests a more accurate representation of input RNA fluctuations when all miRNAs are considered. Normalization methodology that uses global miRNA levels may be valuable compared with a single reference gene.

In order to develop a reliable and reproducible miRNA based test for clinical use as a disease biomarker, the influence of blood processing needs to be clarified. miRNAs have promise as disease biomarkers and in this study we compared Nanostring quantitation of miRNA in serum and plasma samples. Although detection of miRNA in serum may be affected by coagulation, our results indicate the need for careful validation in analyzing plasma samples for discovery studies using this platform. Recognition of the potential variations and impact of choice of blood sample and other pre-analytical factors will help guide future studies aimed at discovery and application studies using this platform.

## Supporting information

S1 Dataset Study data. (TXT)
